# Radiotherapy—the not-so-insignificant contributor to cancer-associated venous thrombosis

**DOI:** 10.1016/j.rpth.2025.102985

**Published:** 2025-07-23

**Authors:** Gerard Gurumurthy, Jacob Miller, Marc Carrier, Alok Khorana, Jecko Thachil

**Affiliations:** 1 The University of Manchester, Manchester, UK; 2 Department of Radiation Oncology, Taussig Cancer Institute, Cleveland Clinic, Cleveland, Ohio, USA; 3 Department of Medicine, University of Ottawa, The Ottawa Hospital Research Institute, Ottawa, Ontario, Canada; 4 Department of Hematology and Medical Oncology, Taussig Cancer Institute, Cleveland Clinic Foundation, Cleveland, Ohio, USA; 5 MAHSC Professor, The University of Manchester, Manchester, UK

**Keywords:** cancer, cancer-associated venous thrombosis, radiation therapy, radiotherapy, venous thromboembolism

## Abstract

Venous thromboembolism is a well-established complication in patients with cancer and a leading cause of morbidity and mortality in these subjects. However, the role of radiotherapy in cancer-associated venous thromboembolism (CAT) remains less clearly defined. The incidence of CAT in this population varies widely, with several large-scale studies suggesting an association. Although management of CAT in this population follows standard guidelines, less is known about the appropriateness of thromboprophylaxis in patients with different types of cancer. Patients with cancer undergoing radiotherapy may also be at increased risk of bleeding, which may be further worsened by anticoagulation. A multidisciplinary approach integrating hematology and oncology expertise is essential in this setting. Further research is needed to establish standardized protocols and predictive models to identify those at risk of thrombosis and bleeding while on anticoagulation.

## Essentials

• RT’s impact on CAT risk remains unclear.
• We reviewed the incidence of CAT, its mechanisms, and management in RT-treated cancer patients.
• RT is associated with an increased risk of CAT in selected populations.
• Thromboprophylaxis is not routine but may benefit high-risk RT patients under expert guidance.

## 1 Introduction

Venous thromboembolism (VTE) is a recognized complication in patients with cancer and represents a leading cause of morbidity and mortality in this population [1,2]. Cancer is associated with a prothrombotic state that elevates VTE risk by 4- to 7-fold compared with noncancer patients [[3], [4], [5]]. This risk is multifactorial; certain cancers, such as pancreatic and gastric malignancies, are highly thrombogenic, while additional factors include advanced tumor stage, a previous history of VTE, obesity, and prolonged immobility [[6], [7], [8], [9]]. Mechanistically, malignancies drive a hypercoagulable state through the release of procoagulant substances, the generation of inflammatory cytokines, and direct endothelial injury [9]. Standard management of cancer-associated VTE (CAT) involves prophylactic anticoagulation for high-risk individuals and therapeutic anticoagulation for those with established thrombosis. This is commonly achieved through low-molecular-weight heparins (LMWHs) or direct oral anticoagulants (DOACs). This narrative review aims to describe the association between radiotherapy (RT), in its various forms, and VTE, and to elucidate the underlying mechanisms.

## 2 Methods

### 2.1 Search strategy and selection criteria

We searched MEDLINE, Embase, and Ovid for publications in English using the following keywords and Medical Subject Headings: “venous thromboembolism” OR “VTE” OR “deep vein thrombosis” OR “pulmonary embolism” AND “radiotherapy” OR “radiation” OR “SBRT” OR “stereotactic” AND “cancer” OR “malignancy” OR “oncology.” Titles and abstracts were initially screened to identify studies relevant to VTE in the setting of RT. Full texts of potentially eligible articles were reviewed. Reference lists of selected papers were manually examined to identify additional relevant studies. Studies were selected if they provided epidemiological data, mechanistic insights, diagnostic approaches, or clinical guidance on risk stratification and management of VTE in the context of RT for patients with cancer.

## 3 Results and Discussion

### 3.1 RT modalities

RT is a targeted therapeutic modality that utilizes ionizing radiation to induce DNA damage in malignant cells, leading to tumor cell apoptosis while minimizing the effects on surrounding normal tissue [10]. It is often integrated with other treatment modalities to enhance therapeutic efficacy. Chemoradiotherapy, for example, involves the combination of chemotherapy and RT. This can be delivered concurrently, where chemotherapy and RT are administered at the same time to achieve a radio-sensitizing effect that increases tumoricidal effect, or sequentially, where one treatment follows the other.

RT can be broadly divided into external beam RT (EBRT) and brachytherapy. EBRT uses beams generated outside the patient, typically from a linear accelerator, and encompasses multiple modalities. Examples include 3-dimensional conformal RT, which relies on computed tomography (CT) imaging for precise tumor localization and delineation of normal tissues. This approach delivers radiation to the gross tumor volume plus margins for microscopic extension (clinical target volume) and setup variations (planning target volume) [11]. Another modality is intensity-modulated RT, which uses inverse planning software and computer-controlled modulation of beam intensity to sculpt dose distributions that spare normal tissue more effectively. Lastly, stereotactic body RT (SBRT) delivers high doses of radiation (up to 34 Gy per fraction) in a highly focused manner over a limited number of sessions (hypofractionation). SBRT is predominantly used in early-stage non–small cell lung cancer, prostate cancer, and in cases of oligometastatic disease [12]. Brachytherapy, by contrast, involves placing radioactive sources within or near the tumor, either with an interstitial, intracavitary, or superficial technique [13]. The close proximity of these sources to the tumor ensures a high local dose with a rapid fall-off to surrounding normal structures.

### 3.2 RT modalities and VTE

RT may induce vascular endothelial injury and local inflammation, potentially exacerbating the prothrombotic effect in patients with cancer [14,15]. In the context of chemoRT, the concurrent administration of chemotherapy further exacerbates this risk by adding systemic cytotoxic effects and additional endothelial injury [16]. The complex interplay of these treatment modalities means that the thrombotic risk in these patients may be higher than when any single modality is used alone, yet the contribution of RT to VTE is often not discussed. Given the long-term complications of VTE [17,18], efficient diagnosis and management of VTE is warranted in those with cancer, given their increased risk.

### 3.3 Incidence and risk factors

The incidence of VTE in RT is not well-defined in the literature (Table). Some suggest a weak correlation between the incidence of VTE and RT [20,24,25]. A prospective radiation-induced thrombosis (RIT) study of 400 patients receiving curative RT for various cancers reported a 6-month cumulative VTE incidence of approximately 2.0% [20]. However, only half of these events occurred during the RT course, suggesting that the absolute risk attributable directly to RT is likely lower. Notably, none of the patients in this cohort received routine thromboprophylaxis.

**Table:** Incidence of venous thromboembolism in various cancer types and radiotherapy modalities.

Study	Cancer types	RT modality	Sample size, *N*	Follow-up, mo	Cumulative VTE incidence reported, %	Estimated incidence rate (per 100 patient-years)
Temraz et al. [19]	Breast, lung, colon, ovarian	Not specified	361	6	9.1	18.2
Daguenet et al. [20]	Breast, prostate, head and neck, cervix, gastrointestinal, lung, CNS, and bladder	EBRT and brachytherapy	401	6	2.0	4.0
Cherkashin and Berezina [21]	Brain	3D conformal RT	165	Not specified	6.1	Not calculable
	Abdominal, pelvic, lung, and breast	Conventional RT	158		2.5	
Ohsumi et al. [22]	Breast	Not specified	431	12	0.2	0.2
Ezer et al. [23]	Lung	SBRT	362	27 (median)	6.9	3.07
Yuk et al. [24]	Endometrial	EBRT	311	6	1.0	2.0
		Brachytherapy	315		0.32	0.64
		Various with chemotherapy	17		0.0	0.0
Bosco et al. [25]	Prostate	EBRT	6232	4.6 (mean)	1.6	4.2
		Brachytherapy	3178	5.1 (mean)	1.4	3.3

3D, 3-dimensional; CNS, central nervous system; EBRT, external beam radiotherapy; RT, radiotherapy; SBRT, stereotactic body radiotherapy; VTE, venous thromboembolism.

However, other large-scale studies suggest that RT can augment VTE risk under certain conditions. A subanalysis of the Comparison of Methods for thromboembolic risk assessemnt with clinical Perceptions and Awareness in real life patients (COMPASS)-CAT study, a prospective trial of 1076 ambulatory cancer patients, identified RT as an independent risk factor for VTE in multivariate analysis in their derived risk assessment model (hazard ratio, 2.47; 95% CI, 1.47-4.12; *P* = .001) [19]. RT-associated VTE risk was notably higher in breast cancer patients [19]. However, the 1-year incidence of VTE in RT-treated breast cancers in the subgroup analysis of the Cancer-VTE study was 0.2% (hazard ratio, 0.31; 95% CI, 0.04-2.70) [22]. The role of RT and VTE in various tumor types is therefore not well defined in the literature.

A retrospective study of cancer patients treated with outpatient RT or chemotherapy was conducted to assess the impact of RT on the risk of CAT relative to chemotherapy. In the cohort of 487 patients, 165 received 3-dimensional conformal RT for brain tumors or metastases (10 VTEs, 6%), 158 had RT to body sites (4 VTEs, 2.5%), and 164 underwent chemotherapy alone as a control group (4 VTEs, 2.4%). Overall, EBRT in group 1 (brain tumors or metastases), but not group 2 (body sites), carried a significantly increased risk of VTE compared with chemotherapy, with a risk difference of 5% [21]. Given the observational nature of this series, it is uncertain whether RT or other potential confounders (eg, tumor site and patient factors) contributed to excess VTE risk.

Large registry data have also yielded associations between RT and VTE. An analysis from the Registro Informatizado de la Enfermedad ThromboEmbolica (RIETE) registry, comprising approximately 9300 patients with cancer and VTE, reported that 13% of patients had been receiving RT at the time of diagnosis [26]. Patients undergoing RT demonstrated a higher rate of pulmonary embolism (PE) recurrences and an increased incidence of cerebral hemorrhagic complications. A multivariate analysis adjusted for cancer site (including central nervous system tumors) confirmed that RT remained independently associated with a higher risk of cerebral bleeding, but not PE recurrence.

Comparison between curative and palliative intent RT is challenging. This is because few studies focus solely on palliative cohorts, and most mixed-intent analyses do not disaggregate VTE rates by intent. Attributing observed differences in VTE incidence to radiation dose or field alone is also fraught with confounding from disease burden and histology, palliative vs concurrent or sequential systemic therapies, and patient performance status. In the fully curative RIT cohort, the 6-month VTE incidence was 2% compared with 9.1% in the mixed-intent COMPASS-CAT population [19,20]. In a retrospective cohort of 2707 patients managed by a specialist oncology palliative care team, the overall VTE prevalence was 22.2% [27], of which only 6.2% occurred during specialist-led palliative care. Treatment modalities were not specified in the study. Thus, the reference rate of 6.2% is useful merely as a benchmark. Until further large prospective studies stratify by RT intent, delineating true VTE risk differentials remains elusive.

SBRT delivers highly focused high-dose radiation in fewer fractions. High-dose irradiation to a localized area may, in theory, cause intense endothelial injury in the irradiated field, which predisposes to VTE. Data specific to SBRT and VTE are limited. A prospective study of 110 patients with lung cancer treated with SBRT found no significant change in coagulation markers [28]. SBRT did not increase thrombin generation or platelet aggregation, indicating no prothrombotic shift in the immediate posttreatment period. In that cohort, a subset did develop VTE during follow-up, but SBRT was not associated with any measurable hypercoagulability trend, yet, one study determined that the rates of thromboembolic events were no different in a group of early-stage non–small cell lung cancer treated with SBRT vs limited resection [23]. Overall, the incidence of RT and VTE remains ill-defined.

### 3.4 Mechanisms contributing to VTE

#### 3.4.1 Endothelial injury and platelet aggregation

**Figure 1:**
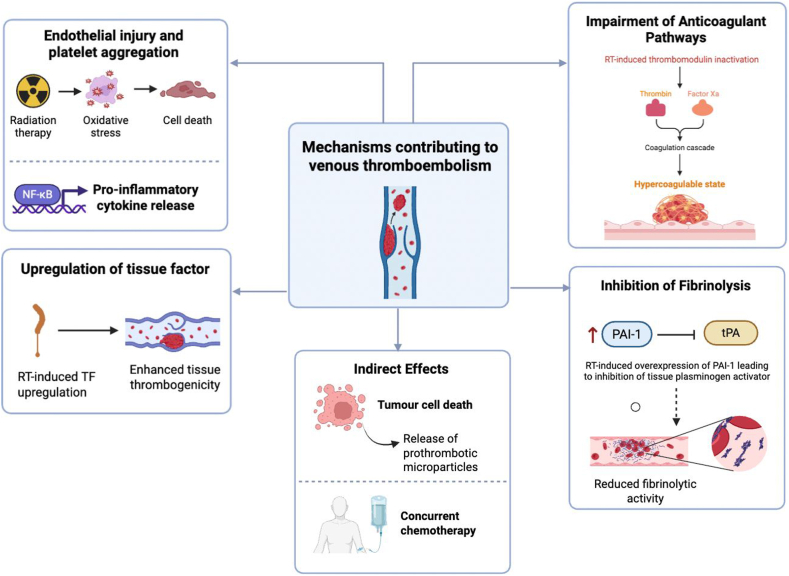
Mechanisms contributing to venous thromboembolism following radiotherapy (RT). RT promotes a prothrombotic state through multiple direct and indirect pathways. Endothelial injury, oxidative stress, and proinflammatory cytokine release activate nuclear factor κB (NF-Κb) and enhance platelet aggregation. RT-induced upregulation of tissue factor (TF) increases tissue thrombogenicity. RT also impairs natural anticoagulant pathways by inactivating thrombomodulin, leading to a hypercoagulable state. Concurrently, fibrinolysis is inhibited via upregulation of plasminogen activator inhibitor-1 (PAI-1), reducing tissue plasminogen activator (tPA) activity. Indirect mechanisms include tumor cell death and concurrent chemotherapy, both of which contribute to the release of prothrombotic microparticles. Collectively, these mechanisms heighten the risk of venous thromboembolism in patients undergoing RT. (Figure made using BioRender).

Multiple mechanisms may explain how RT promotes a prothrombotic state (Figure 1). Radiation induces direct endothelial cell injury and inflammation within irradiated vascular beds [29]. It also triggers the release of inflammatory cytokines and procoagulant molecules, fostering a localized prothrombotic environment [30]. Specifically, radiation has been associated with increased expression of tissue factor (TF), activation of nuclear factor κB pathways, and elevated levels of activated factor VIII, circulating D-dimer, von Willebrand factor (VWF), and platelet activation [30,31]. For instance, in human umbilical vein endothelial cells, acute or fractionated doses of 20 Gy resulted in elevated VWF release, while in rat heart models, VWF deposition increased at 15 to 20 Gy, 3 to 6 months postirradiation [32]. Collectively, these lead to platelet adhesion and aggregation [33].

#### 3.4.2 Upregulation of TF

*In vitro* studies suggest that the upregulation of TF expression, the primary initiator of the extrinsic coagulation pathway, is also implicated [34]. One study found that RT increases TF expression on endothelial cells and monocytes, particularly at doses between 20 Gy and 40 Gy, with significant increases observed on days 3 and 7 postirradiation [35]. It was noted that TF upregulation began from day 1 postirradiation, suggesting an immediate thrombogenic effect from RT. This upregulation, alongside inflammation and apoptosis, enhances tissue thrombogenicity, thereby contributing to thrombosis.

#### 3.4.3 Impairment of anticoagulant pathways

RT may downregulate natural anticoagulant systems through the inactivation of thrombomodulin (TM). TM is an endothelial receptor with anticoagulant and anti-inflammatory properties [36]. Studies in cell-free systems demonstrate that ionizing radiation oxidizes a specific methionine residue (Met388) in TM [37]. This reduces its ability to activate protein C, thus diminishing anticoagulant activity. The inactivation contributes to a hypercoagulable state.

#### 3.4.4 Inhibition of fibrinolysis

RT may induce sustained expression of plasminogen activator inhibitor-1, which inhibits tissue plasminogen activator, reducing fibrinolytic activity. A study on irradiated microvascular veins harvested from 15 irradiated patients found increased plasminogen activator inhibitor-1 gene expression, explaining adverse effects like microvascular occlusion postradiation [38]. This sustained inhibition contributes to persistent thrombus formation, heightening VTE risk.

#### 3.4.5 Indirect effects

Tumor cell death may lead to the release of prothrombotic microparticles, which can promote thrombogenesis [[39], [40], [41]]. Additionally, RT’s thrombotic association may be mediated by concurrent chemotherapy or the postoperative state in combined treatment regimens. One analysis suggested that the apparent effect of RT on CAT could be partially confounded by cancer type and therapy context [19].

### 3.5 Diagnosis of VTE during RT

The diagnosis of VTE in cancer patients may be complicated by overlapping syndromes. Conversely, unexplained symptoms during RT should not be prematurely attributed to radiation side effects without considering VTE. For example, radiation pneumonitis often presents with cough and dyspnea. Guidelines on pneumonitis, therefore, suggest that PE is a crucial differential diagnosis that should be considered [42,43]. Any acute decompensation should prompt workup for PE [44]. Additionally, radiation to the pelvis may lead to chronic radiation injury, and patients in this cohort show a 15% rate of “suspicious” symptoms that warrant ultrasound, leading to a 5% incidence of lower extremity deep vein thrombosis [45]. As such, when new cardiopulmonary or extremity symptoms appear during or after RT, the threshold for Doppler ultrasound or CT pulmonary angiography (CTPA) should be the same as, if not lower than that in nonirradiated patients with cancer. A multidisciplinary approach integrating radiation oncologists, hematologists, and radiologists is crucial to ensure appropriate diagnostic pathways are followed. Ultimately, a high index of suspicion, thorough symptom evaluation, and appropriate imaging are essential for the timely recognition and diagnosis of VTE in patients undergoing RT.

The diagnostic approach to suspected VTE in RT patients follows standard protocols. However, there are some cancer-specific considerations. While D-dimer testing has high sensitivity and is typically used to rule out VTE in low-risk populations, its utility is significantly limited in patients with cancer due to elevated baseline levels related to malignancy and inflammation, reducing its specificity and negative predictive value [[46], [47], [48]]. As a result, D-dimer should be interpreted with caution and is not a reliable tool to exclude VTE in cancer patients. Additionally, D-dimer may be elevated postirradiation due to radiation-induced coagulopathies and endothelial injury, as explored earlier [49,50]. Consequently, imaging should not be delayed in lieu of D-dimer testing. Conversely, small case series have shown that some patients with cancer and radiologic evidence of PE can present with normal D-dimer, and therefore, a normal D-dimer should be interpreted with caution [51]. Reasons for this may include low clot burden, impaired fibrinolytic activity due to tumor-secreted proteolytic factors, and consumptive coagulopathy [[51], [52], [53]]. The preferred diagnostic modalities remain ultrasonography for suspected deep vein thrombosis and CTPA for suspected PE, both of which maintain high diagnostic performance in patients with cancer [54].

### 3.6 Post-RT artifacts

RT can generate vascular and parenchymal changes in the irradiated lung that may resemble intravascular filling defects on CTPA. Although true cancer-associated PE remains a major concern, several recent imaging series show that a minority of the artifacts detected during post-RT surveillance represent indolent, *in situ* thrombi that behave very differently from acute embolic disease. The largest dedicated series to date identified 27 cases of *in situ* pulmonary artery thrombosis (PAT) after thoracic RT [55]. Radiation-induced lung fibrosis was present in the ipsilateral lung in all patients. All thrombi lay entirely within the high-dose treatment volume and none embolized elsewhere during a median follow-up of 22 months, even when anticoagulation was withheld.

Nonocclusive *in situ* PAT within the radiation field may be invariably eccentric, form an obtuse rather than an acute angle with the vessel wall, spare the distal caliber of the artery, and be surrounded by established radiation fibrosis that conforms to the planning target volume [55]. In the above-mentioned study [55], thrombi developed several months after treatment (median, 675 days) and remained stable or slowly regressed, unlike conventional PE.

A repeat CTPA with tighter bolus timing may clarify the findings. Alternatively, a ventilation-perfusion scan remains an excellent arbiter as true emboli generate wedge-shaped perfusion defects, whereas *in situ* PAT and beam hardening do not alter perfusion. These artifacts mostly do not require any further intervention but should be revisited if a patient develops cardiopulmonary symptoms further down the line.

### 3.7 Thromboprophylaxis during RT: when do we use it?

There is no evidence that all RT patients should receive prophylactic anticoagulation, as available studies demonstrate a mixed level of VTE incidence attributable to RT alone. Investigators in the prospective RIT study concluded that routine thromboprophylaxis in the setting of curative RT was not warranted due to the lack of a clearly defined high-risk population [20]. Thus, blanket prophylaxis for all RT outpatients is not recommended. However, studies examining thromboprophylaxis during chemoradiation and brachytherapy suggest it may reduce the incidence of thromboembolic events [56]. Selected patients with multiple risk factors for VTE may therefore benefit from thromboprophylaxis. One study identified that those aged >50 years, patients receiving anthracycline chemoRT, and hormonal therapy were factors that increased VTE risk in RT [19]. However, given the variability in risk across cancer types and the lack of standardized risk assessment models incorporating RT, the authors concluded that more research is needed to define prophylaxis strategies in this patient group [19]. Additionally, thromboprophylaxis may be considered for those undergoing chemoRT with a Khorana score ≥2 [57]. In summary, no current guidelines support routine thromboprophylaxis in all RT patients, and its use should be limited to carefully selected individuals until stronger evidence becomes available.

If thromboprophylaxis is to be considered, apixaban has shown efficacy for prophylaxis in CAT [58]. It should be utilized around the time of RT; the timing should be optimized to provide protection when the thrombotic risk is highest. Available data suggest that VTE events in RT patients occur both during treatment and in early posttreatment [20]. Therefore, initiating prophylaxis at the start of RT (or a few days prior) and continuing for a short period post-RT (eg, 2-4 weeks) may be a reasonable strategy in high-risk cases. There is currently no defined optimal duration for prophylaxis in RT patients, and decisions must therefore be made based on individualized risk assessment.

Importantly, prophylaxis should be avoided in patients at high risk of bleeding. Those with active gastrointestinal ulceration, brain metastases, severe thrombocytopenia, or recent tumor-associated bleeding (hemoptysis, hematuria, or vaginal bleeding) should not receive prophylactic anticoagulation, as the potential for harm may outweigh the benefits.

### 3.8 Management of VTE in RT

There is a lack of evidence in the literature to define the optimal anticoagulation strategy of VTE in those undergoing RT. The management of VTE in this patient population, therefore, aligns with the general principles of treating CAT. According to current guidelines from the American Society of Clinical Oncology, LMWH or DOACs are recommended for at least 3 to 6 months, with extended treatment considered for patients with ongoing cancer activity or persistent risk factors [59]. Given that many patients remain at elevated risk due to ongoing malignancy, extended anticoagulation is often warranted. The British Society for Haematology recommends anticoagulation therapy >6 months for CAT and active cancer [60].

Therapeutic anticoagulation should be initiated promptly upon VTE confirmation unless contraindications exist. Historically, LMWH was the preferred agent for CAT due to evidence of superiority over warfarin in reducing recurrence risk [61]. However, more recent trials have demonstrated the efficacy and safety of DOACs, making them convenient alternatives [62]. DOACs are increasingly used in clinical practice over LMWH due to their lower risk of VTE recurrence and comparable risk of major bleeding, as determined in a meta-analysis of 6 randomized trials [63].

**Figure 2:**
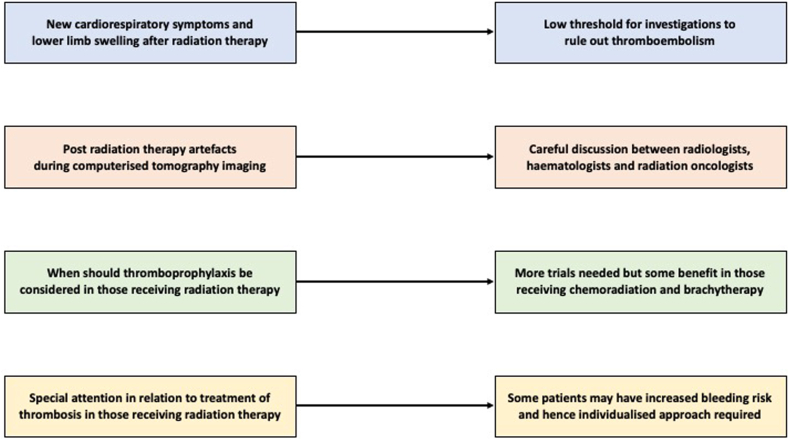
Clinical considerations for venous thromboembolism in patients receiving radiotherapy (RT). New or unexplained cardiopulmonary or lower limb symptoms in the post-RT setting warrant prompt evaluation to exclude venous thromboembolism. Post-RT imaging artifacts may mimic thromboembolism and require multidisciplinary interpretation. Thromboprophylaxis is not routinely recommended given the lack of evidence to support its use. Treatment decisions must balance thrombotic risk with bleeding potential, necessitating an individualized approach.

Close collaboration between hematologists and radiation oncologists is essential when initiating anticoagulation during RT (Figure 2). In most cases, RT can proceed uninterrupted alongside anticoagulation, but careful monitoring is required. However, there are specific scenarios that necessitate additional precautions. First, patients with brain metastases or receiving cranial irradiation are at an increased risk of intracranial hemorrhage when anticoagulated, as reported in the RIETE registry study [26]. If a patient is undergoing whole-brain RT or stereotactic radiosurgery, the bleeding risk must be weighed against the benefits of anticoagulation. In select cases, inferior vena cava filter placement may be considered as an alternative to systemic anticoagulation, though current guidelines generally discourage filters due to reported lower efficacy in recurrent VTE prevention in the setting of brain metastases [64]. Second, RT to large hematopoietic sites, such as the pelvis, especially with concurrent chemotherapy, can cause significant cytopenias, including chemotherapy-induced thrombocytopenia [[65], [66], [67], [68]]. If platelet counts fall < 50 × 10^9^/L, full-dose anticoagulation may need to be adjusted or temporarily paused to mitigate bleeding risk. In such cases, the treating team should be aware of the possible complications, and a multidisciplinary approach is required to balance recurrent VTE prevention with safety while ensuring that RT fractions can still be delivered effectively without undue interruptions. Lastly, DOAC metabolism involves P-glycoprotein and CYP3A4 pathways, creating potential interactions with chemotherapy agents [[69], [70], [71]]. A thorough medication review should be conducted, and LMWH may be preferred if major interactions are present.

### 3.9 Incidental VTE

Patients with cancer undergoing RT frequently require periodic CT imaging for treatment planning or tumor response assessment, leading to incidental VTE detection [72]. Incidental VTE findings on routine CT scans, such as subsegmental PEs or pelvic vein thromboses, are common [73]. Moreover, most treatment planning CT scans are not reviewed by diagnostic radiologists, highlighting the importance of careful review by the treating radiation oncologist for incidental VTE. The prevalence of incidental PE in cancer patients is not well-defined but is believed to be up to 15% [[74], [75], [76]]. One prospective registry study of cancer patients noted that incidental VTE formed 27% of all thromboembolic cases in their cohort [77]. Studies demonstrate that incidental asymptomatic PEs have a similar anatomic distribution to symptomatic PEs [73,78]. Studies have also shown a similar prognosis for incidental and symptomatic VTE in patients with cancer [[79], [80], [81]]. Incidental VTEs were included in trials assessing DOACs and LMWH for the acute management of CAT (32% in HOKUSAI VTE Cancer [62], 52% in Select-D [82], and 20% in CARAVAGGIO [83]) and, therefore, should be treated with conventional approaches [84]. Treatment with anticoagulation using LMWH and DOACs should be commenced [85]. The optimal duration of anticoagulation is not well-defined, but a shorter duration may be considered in those with an increased risk of bleeding [84].

### 3.10 Risk stratification models for VTE and bleeding risk

Accurate risk prediction is essential for determining which cancer patients undergoing RT are likely to develop VTE or experience bleeding complications while on anticoagulation. To aid clinicians further, risk stratification tools identify patients who may benefit from thromboprophylaxis while minimizing unnecessary exposure to anticoagulation-related bleeding risks. The most widely used risk model for CAT is the Khorana score, which incorporates cancer type, platelet count, hemoglobin (or erythropoietin use), leukocyte count, and body mass index to stratify ambulatory chemotherapy patients into low-, intermediate-, and high-risk VTE categories [86]. A score ≥2 has been shown to predict better outcomes when pharmacologic thromboprophylaxis is used; therefore, patients with such scores are recommended for prophylactic anticoagulation [58,87]. However, the Khorana score was developed for chemotherapy-treated patients and does not explicitly account for RT, yet, it has been shown to have predictive ability for both VTE incidence and survival in a mixed chemotherapy and RT retrospective cohort [57]. It can therefore guide clinicians with risk stratification of an RT individual in the absence of an RT-specific tool. Additional models, such as the COMPASS-CAT score (which includes prior VTE, body mass index, and hormonal therapy), Electronic Health Record (EHR)-CAT (which uses components of the Khorana score with additional variables such as cancer staging, systemic therapy class, history of VTE, and race), and the Vienna CATS model (which integrates D-dimer and P-selectin levels), are externally validated tools that may provide useful risk stratification, although they have not been specifically validated in an RT population [[88], [89], [90], [91], [92], [93]].

On the other hand, bleeding risk assessment is crucial when deciding on prophylaxis or long-term anticoagulation. Patients with cancer are noted to be at increased risk of bleeding while on anticoagulation therapy [[94], [95], [96]]. The need for anticoagulation, therefore, needs to be weighed against the bleeding risk induced by this treatment. The CAT-BLEED model is one example of a bleeding risk assessment tool designed specifically for CAT, but its performance is moderate, and it has not been externally validated [97]. Currently, there remains no externally validated risk assessment model for VTE and RT specifically. The VTE-PREDICT and RIETE models were designed to estimate both VTE recurrence and bleeding risk in patients who have completed initial anticoagulation [98,99]. While it was initially validated in a noncancer population, its principles may be applied to patients with cancer undergoing RT to balance the risk-benefit ratio of prolonged anticoagulation. However, one study examining both models in a cohort of 110 patients with cancer determined that neither model could highly predict bleeding events in this group [100]. This reflects the clinical reality that many patients with cancer have multiple risk factors, making it difficult for a linear model to single out a low-risk subset. In such a setting, clinical judgment and a pragmatic classification by cancer type might be as good as formal scoring systems for gauging bleeding risk.

There are some considerations that may guide clinicians when assessing the risk of bleeding while on anticoagulation in the setting of CAT. A secondary analysis of the Cancer-related VTE Anticoagulaton Strategies (CANVAS) trial, aimed at identifying factors of bleeding in patients with cancer, concluded that both serum albumin < 3.5 g/dL and metastatic disease were independent predictors [101]. In clinical practice, other high bleeding risk features include primary or metastatic brain tumors, gastrointestinal, thoracic, or gynecologic cancers with high-risk lesions, severe thrombocytopenia, and recent surgery. The RIETE registry analysis highlighted the increased risk of intracranial hemorrhage in RT patients who were anticoagulated [26]. Also, one meta-analysis identified an increased bleeding risk associated with DOACs compared with LMWH [102]. In those with a moderate bleeding risk who require anticoagulation, LMWH may be a viable consideration and is endorsed by several guideline bodies. Given the lack of a discriminative model for bleeding risks, the factors listed should be considered when making anticoagulation decisions in RT patients to assess their risk of bleeding.

## 4 Conclusion

While patients with cancer already face an elevated baseline thrombotic risk, RT may contribute particularly to high-risk subgroups. Management strategies should emphasize early recognition of VTE, evidence-based anticoagulation therapy, and careful consideration of risks for both VTE occurrence and bleeding while on anticoagulation. Thromboprophylaxis should not be routinely administered to all RT patients but may be considered in highly select individuals with high-risk features, though specific recommendations remain an area for future research.
